# Severe Symptomatic Anemia as a Rare Initial Manifestation of Type 3 Polyglandular Autoimmune Syndrome: A Case Report

**DOI:** 10.7759/cureus.75800

**Published:** 2024-12-16

**Authors:** Hugo Goncalves, Francisco De Oliveira Simões, Rosa Sá, Bárbara Fraga Campos, Rui M Domingues, Narciso Oliveira, Teresa Pimentel

**Affiliations:** 1 Division of Rheumatology, Unidade Local de Saúde de Braga, Braga, PRT; 2 Division of Internal Medicine, Unidade Local de Saúde de Braga, Braga, PRT; 3 Division of Oncology, Unidade Local de Saúde de Braga, Braga, PRT; 4 Internal Medicine, Hospital de Braga, Braga, PRT

**Keywords:** autoimmune gastritis, autoimmune polyglandular syndrome, hashimoto's disease, poly-autoimmunity, vitiligo

## Abstract

Autoimmune polyglandular syndrome type 3 (APS-3) is an uncommon condition marked by autoimmune thyroid disease (ATD) linked with other autoimmune issues, excluding Addison’s disease. We report a case of a 41-year-old man who was hospitalized due to exhaustion and macrocytic anemia, later diagnosed with APS-3, which included Hashimoto’s thyroiditis, pernicious anemia resulting from autoimmune gastritis, and pre-existing vitiligo. Diagnostic results indicated positive intrinsic factor antibodies, a gastric biopsy compatible with gastritis, elevated thyroid peroxidase antibodies, and significant findings from a thyroid ultrasound. Treatment included parenteral vitamin B12, which improved anemia, while for mild hypothyroidism, a watchful waiting approach was taken. This case report highlights the importance of conducting a systematic evaluation of anemia in the diagnosis and management of APS-3. Although anemia is not typically recognized as a characteristic feature of APSs, it can present as pernicious anemia secondary to autoimmune gastritis. These anemias can be significant and severely limiting, often causing pronounced symptoms that greatly impact patients’ daily lives, further emphasizing the need for timely diagnosis and appropriate management.

## Introduction

Autoimmune polyglandular syndrome type 3 (APS-3) is one of the three variants of APS, which are defined on the basis of the number of glands affected [1]. APS-1 is one type of polyglandular syndrome that affects multiple glands and is passed on in an autosomal recessive manner. Importantly, it is associated with a triad of chronic tonsil candidiasis, hypoparathyroidism, and adrenal dysfunction. APS-2 is more common with a triad of Addison disease, autoimmune thyroid disease, and type 1 diabetes. APS-3 is a type of organ-specific autoimmune disorder in which autoimmune thyroid disease (ATD) occurs in combination with other immune-mediated disorders such as pernicious anemia, vitiligo, and alopecia but with the absence of adrenal insufficiency [1,2]. Because the initial symptoms in such patients are often non-specific, they can be misdiagnosed or remain undiagnosed, necessitating a coordinated investigation into the involvement of multiple organ systems.

Anemia can be a significant problem for patients suffering from APS-3, particularly for those with pernicious anemia due to autoimmune gastritis. It is a type of anemia that occurs due to the destruction of gastric parietal cells, which results in a deficiency of vitamin B12 due to a lack of intrinsic factors being produced. If not treated, it can be disabling, leading to the development of clinical signs and symptoms like low tolerance for physical activity, dyspnea, and thoracic pain. 

We describe in detail the clinical presentation, diagnostic workup, and management of a patient with APS-3. The report emphasizes the relationships between associated autoimmune processes in this condition.

## Case presentation

A 41-year-old male computer engineer, who lives in an urban apartment, was admitted to the emergency department due to fatigue for the last two months and chest pain for the last week. The patient’s condition deteriorated over the period of one month due to increasing fatigue and over the week prior to the hospitalization he had been exhausted and bedridden. The patient described his pain as sharp and pleuritic in character but not triggered by movement. He had no fever, night sweats, weight loss, joint pains, and no sinonasal, respiratory, or gastrointestinal complaints. Vitiligo was diagnosed two years earlier but no other significant medical conditions or chronic medication.

On examination, the patient was awake, oriented, and hemodynamically stable with a blood pressure of 136/65 mmHg and a heart rate of 87 bpm. The patient’s mucosas were pale but moist, and there were no lymph nodes or organ enlargement. There were no significant findings in cardiac and respiratory examinations. Neurological examination was unremarkable.

Hematological analysis indicates the presence of macrocytic anemia with a hemoglobin concentration of 6.2 g/dL, mean corpuscular value (MCV) was 99.1 fL, and reticulocyte production index was noted as only 0.7%, thereby suggesting poor red blood cell production. Vitamin B12 was found to be low, 51 pg/mL, which was compatible with a vitamin B12 deficiency. The patient was admitted for the investigation of macrocytic anemia and subclinical hypothyroidism. The detailed laboratory findings at the emergency department are presented in Table 1.

**Table 1:** Detailed laboratory findings at the emergency department. Detailed laboratory findings at the emergency department. MCV: mean corpuscular value; CHCM: cellular hemoglobin concentration mean; RPI: reticulocyte production index; TIBC: total iron binding capacity; TS: transferrin saturation; TSH: thyroid-stimulating hormone; FT4: free T4

Laboratory test	Laboratory values	Reference values
Hemoglobin	6.2 g/dL	13.5-17.0 g/dL
MCV	104.9 fL	81.8-95.5 fL
CHCM	35.9 g/dL	32.5-34.0 g/dL
RPI	0.7%	<2% meaning inappropriate medullary response
Folic acid	9.4 ng/mL	>5.38 ng/mL
Vitamin B12	51 pg/mL	211-911 pg/mL
Serum iron	261 μg/dL	30-322 μg/dL
Ferritin	232 ng/mL	15-200 ng/mL
TIBC	356 μg/dL	240-425 μg/dL
TS	73%	20-45%
TSH levels	7.839 mμIU/mL	0.4-4.0 mμIU/mL
FT4	1.0 ng/dL	0.8-1.8 ng/dL

After admission, the presence of intrinsic factor antibodies was noted, which suggests the presence of an autoimmune gastritis. Thyroid function tests revealed mild hyperthyroidism with TSH of 7.839 at muIU/mL (normal 0.4-4.0 muIU/mL) and free T4 at 1.0 ng/dL (normal 0.8-1.8 ng/dL). The detailed laboratory findings are presented in Table 1.

A blood smear revealed the presence of anisocytosis, macroovalocytes, and isolated occurrences of basophilic stipplings, which are compatible with megaloblastic anemia. Thoracoabdominopelvic CT scans were normal, as such there was no organ or lymph node enlargement.

A gastric biopsy confirmed a diagnosis of autoimmune gastritis, which, combined with the presence of anti-intrinsic factor antibodies, explained the severe vitamin B12 deficiency, leading to the conclusion of pernicious anemia. The treatment protocol used for this diagnosis consisted of intramuscular hydroxocobalamin 1 mg administered three times per week for two weeks starting at the time of admission, followed by 1 mg once weekly, with subsequent reassessment during follow-up consultations. Hemoglobin concentration increased from 6.2 g/dL to 10.2 g/dL within five days, which helped to resolve most of the symptoms (Table 2).

**Table 2:** Laboratory values at the emergency department; 5 days after parenteral vitamin B12 supplementations; 6 weeks after hospital discharge. RPI (Reticulocyte production index)

Laboratory test	Emergency department	After parenteral vitamin B12	6 weeks after discharge	Reference values
Haemoglobin	6.2 g/dL	10.2 g/dL	14.2 g/dL	13.5-17.0 g/dL
RPI	0.7%	5.6%	3.5%	<2% meaning inappropriate bone marrow response
Vitamin B12	51 pg/mL	1154 pg/mL	> 2000 pg/mL	211-911 pg/mL

**Figure 1:**
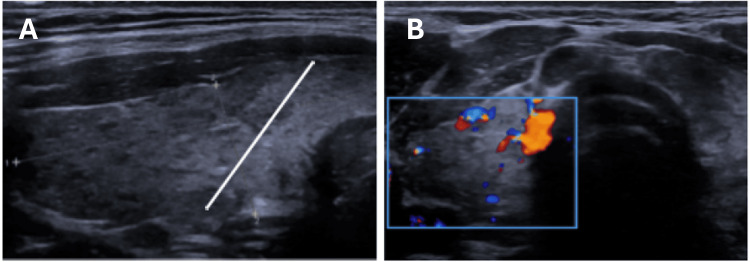
Thyroid ultrasonography Thyroid ultrasonography shows diffusely hypoechoic and heterogeneous echotexture (mainly to the left of the white line, as shown in Image A), with increased vascularization (blue rectangular shape, as shown in Image B).

The anti-thyroid peroxidase antibodies (anti-TPO) were elevated, 830 IU/mL (reference: < 35 IU/mL), corroborating the diagnosis of Hashimoto’s thyroiditis, while anti-thyroglobulin antibodies and TSH receptor antibodies (TRAbs) were negative. Thyroid ultrasonography (Figure 1) showed diffuse parenchymal heterogeneity and hypoechogenicity with areas with increased vascularization, findings in keeping with inflammatory changes caused by Hashimoto’s disease.

Six weeks after discharge, during follow-up, the patient showed significant improvement, with no symptoms and complete resolution of their anemia on laboratory analysis (the laboratory findings six weeks after discharge are presented in Table 2, including comparisons of the laboratory values at admission, at the time of discharge, and six weeks after discharge).

## Discussion

This case illustrates the diagnostic challenges of APS-3 and how it presents with multiple autoimmune manifestations: gastritis, pernicious anemia, Hashimoto thyroiditis, and vitiligo. 

**Figure 2:**
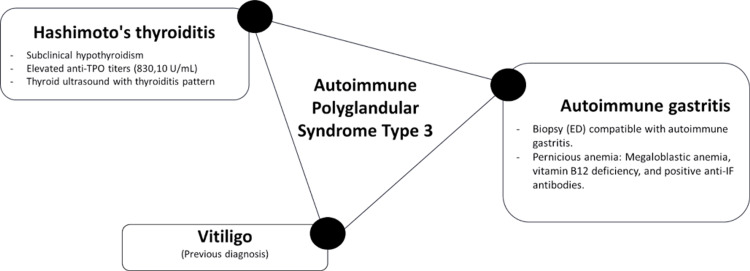
Schematic representation of the clinical presentation of this patient with autoimmune polyglandular syndrome type 3 (APS-3). Illustration created by the authors.

The diagnosis of autoimmune gastritis and subsequently pernicious anemia was corroborated by positive intrinsic factor antibodies, low levels of vitamin B12, and atrophic gastritis. This pathophysiology is rooted in the deficiency of intrinsic factors, which impairs the intestinal absorption of vitamin B12 in the ileum, leading to systemic deficiency and its associated clinical manifestations [3]. Megaloblastic anemia occurs when red blood cell production is impaired due to defective DNA synthesis [4]. Vitamin B12 deficiency is a common cause of this condition, and the poor absorption of vitamin B12 is a critical factor. Extensive hematologic improvement was observed following the initiation of parenteral vitamin B12 supplementation for the successful management of pernicious anemia (Figure 2).

The subclinical status of hypothyroidism, elevated anti-thyroid peroxidase (anti-TPO) antibody levels, and the characteristic features of thyroid ultrasound (Figure 1) of diffusely hypoechoic and heterogeneous echotexture, with increased vascularization, which all pointed to thyroiditis supported the diagnosis of Hashimoto’s disease. Management of the subclinical hypothyroidism (TSH levels were less than 10 μUI/mL and the patient was asymptomatic) was watchful waiting as recommended by the most recent guidelines [5]. 

The coexistence of autoimmune gastritis, pernicious anemia, and Hashimoto's thyroiditis, coupled with a history of vitiligo, strongly suggests a diagnosis of APS-3 (Figure 2).

## Conclusions

This case demonstrates the importance of recognizing APS-3 as a multi-system autoimmune disorder requiring a multidisciplinary approach. Early identification and targeted treatment of individual components, such as pernicious anemia, can lead to rapid symptomatic relief and improved patient outcomes. Long-term follow-up remains essential, both for monitoring disease progression and for identifying new autoimmune manifestations. Close interdisciplinary collaboration is pivotal in ensuring the best outcomes for patients with complex autoimmune syndromes such as APS-3.
